# First dutch experience of the accurate neo self-expanding supra-annular valve for valve-in-valve transcatheter aortic valve implantation

**DOI:** 10.1007/s12471-018-1087-3

**Published:** 2018-02-09

**Authors:** K. H. Soon, N. H. M. Kooistra, M. Voskuil, A. O. Kraaijeveld, P. R. Stella

**Affiliations:** Division of Heart & Lungs, Department of Cardiology, University Medical Center Utrecht, Utrecht, The Netherlands

**Keywords:** Transcatheter Aortic Valve Implantation, Dutch Experience, Bioprosthesis, Balloon Postdilatation, Balloon Predilatation

We describe the first two cases of valve-in-valve (ViV) transcatheter aortic valve implantation (TAVI) using Accurate Neo device in the Netherlands.

A 73-year-old man underwent transfemoral ViV-TAVI for severe stenosis in his 25-mm Carpentier-Edwards Perimount bioprosthesis. After balloon predilatation, a medium-size Accurate Neo was advanced to the bioprosthetic annulus. First, its upper part was released with the upper crowns positioned below the superior margin of the pre-existing bioprosthesis (Fig. 1a; [1]). Next, the device was deployed by releasing the lower crowns without ventricular pacing (Movie-1). The results were excellent.

A 79-year-old man underwent transfemoral ViV-TAVI for severe regurgitation of his 27-mm Mosaic bioprosthesis. A small-size Accurate Neo was deployed with its upper crowns positioned along the eyelets of the bioprosthesis (Fig. 1b). After balloon postdilatation, a paravalvular leak was reduced to trace.

Accurate Neo, a self-expanding supra-annular valve, has the advantages of improved ViV haemodynamics and top-down deployment without pacing. However, it is non-repositionable.

**Fig. 1:**
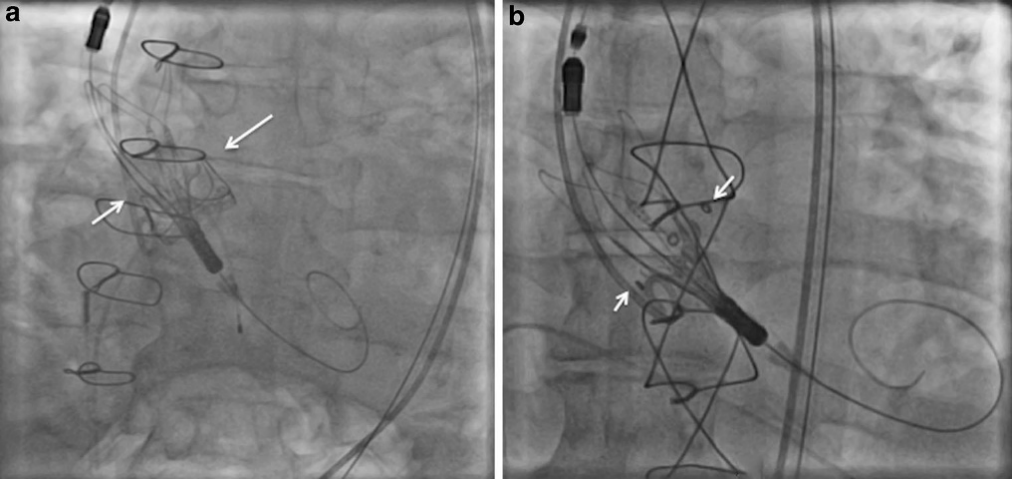
**a** Positioning of an Accurate Neo device inside a Carpentier-Edwards Perimount aortic bioprosthesis with its upper crowns sitting just below the superior margin of the bioprosthesis (*arrows*). **b** Positioning of an Accurate Neo device inside a Mosaic aortic bioprosthesis with its upper crowns positioned along with the eyelets of the bioprosthesis (*arrows*)

## Caption Electronic Supplementary Material

Video 1 Successful deployment of Accurate Neo device in a Carpentier-Edwards Perimount bioprosthesis with no trace of aortic regurgitation

Video 2 Trace of aortic regurgitation of Accurate Neo device in a Mosaic bioprosthesis after balloon postdilatation

## References

[1] Bapak V (2014). Valve-in-valve apps: why and how they were developed and how to use them. EuroIntervention.

